# Family interests and medical decisions for children

**DOI:** 10.1111/bioe.12376

**Published:** 2017-09-13

**Authors:** Paul Baines

**Keywords:** best interests, children, family interests, medical ethics

## Abstract

Medical decisions for children are usually justified by the claim that they are in a child's best interests. More recently, following criticisms of the best interests standard, some advocate that the family's interests should influence medical decisions for children, although what is meant by family interests is often not made clear. I argue that at least two senses of family interests may be discerned. There is a ‘weak’ sense (as the amalgamated interests of family members) of family interests and a ‘strong’ sense (that the family itself has interests over and above the interests of individuals). I contend that there are problems with both approaches in making medical decisions for children but that the weak sense is more plausible. Despite this, I argue that claims for family interests are not helpful in making medical decisions for children.

## 1 INTRODUCTION

When medical decisions are made for children, the justification is usually that the choice is in the child's best interests.^1^ However, the best interests^2^ standard has been criticised^3^ and has led to other suggestions, amongst these that a child's medical decisions should be based on family interests.^4^ However, when family interests are invoked, it is rare that a clear exposition of family interests is given. Sometimes the suggestion is that parents should be involved in making decisions. Sometimes the suggestion is that parents should choose the child's interests^5^ and sometimes the suggestion for family interests is broader than this, extending even to discussion with ‘religious authorities’.^6^ If family interests are to be invoked, there would need to be a clear sense of what is meant.

Contemporary philosophers have turned their attention to groups: Group agency, group autonomy, group intention, group rights and so on, characteristics that are more usually ascribed to a person.^7^ Although these concepts may be clear when applied to individuals, what they mean when applied to groups is not. There are many difficulties to be overcome, not least accounting for the very different sorts of groups that exist (extending from the mob of the French Revolution to the Board of Volkswagen). Claims are made for family autonomy, interests, rights and so on, but recent discussions of groups rarely consider the family. That the family does not appear in discussions of group rights, group agency and group autonomy is surprising, because of the ubiquity of families and their importance in society. This stands in contrast to the frequency with which those discussing families make references to these characteristics. In the absence of a clear characterisation of the terms and in particular what they mean when ‘family’ is attached to them, the claims could be treated as merely metaphorical. However, those that use them do not seem to use them metaphorically. I will concentrate my criticisms on family interests.

In this article, I will demonstrate that different authors in the medical literature have had different conceptions of family interests. I distinguish a weak and strong sense of family interests, arguing that the weak sense of family interests is more defensible, but even so family interests are unhelpful when medical decisions must be made for children.

## 2 FAMILY INTERESTS

When the problems with the best interests standard for children's medical decisions are recognised, some people appeal to the concept of the family's interests: “…what is best, all things considered, for the family.”^8^ Moving to family interests may solve three problems with the individual child's interests. Firstly, the child's best interests’ standard has been criticised for being too demanding. This criticism can be sidestepped by appealing to the family's interests. Secondly, as it is unlikely that an action in the best interests of one child will be in the best interests of all family members, it is unjust to prioritise the interests of one child. If so, we should amalgamate the interests of family members. Thirdly, the concept of family interests recognises interests that parents (and other relatives)^9^ have in their children (and older children have in the well‐being of their relatives). This is all well and good, but family interests are often brought in as a solution to the problems posed by the child's best interests without a clear description^10^. In the absence of a clear conception of family interests, the claim that family interests will allow the resolution of contentious medical decisions is unfounded.

The claim that decisions for children should be based on family interests should be separated from the claim that parents should make a child's medical decisions (a claim for parental authority). Although (following on from the way that individuals are often taken to be the only ones capable of determining their own interests) there is a sense in which the family must select the content of the family interests (the family determines the family traditions, concerns and priorities),^11^ there could also be an ‘objective’ sense of family interests so that those outside the family are at least as well placed as those inside the family to decide how best to maximize family interests (the disadvantages of those outside the family in having less information about the family may be balanced by their detachment). This is analogous to the way that an individual may be mistaken about their own interests. I do not intend to argue whether interests are subjective or objective,^12^ just to argue that a claim that decisions should be based on family interests is separable from the claim that parents should make medical decisions for their child.

The first problem to overcome in arguing for family interests is to understand what is meant by ‘family’.

### 2.1 The lack of a clear definition of a family

The family, and conceptions of a family, are not static. Nussbaum notes “…Family is itself a political institution that is defined and shaped in fundamental ways by laws and social institutions.”^13^ This is demonstrated by marked changes in families over time and in different societies. Single mothers leading families, and same sex families are two examples of recent changes.

Until a few years ago, single unmarried mothers were stigmatised as ‘fallen women’, and coerced into allowing adoption for their children, having been persuaded that adoption was best for both mother and child.^14^ Now, women commonly lead single parent families. Twenty per cent of NHS Trusts fund IVF treatment for single women.^15^ Private IVF clinics target single women.^16^ Single motherhood, once humiliating for the mother, is now a deliberate choice. Another example is that homosexuality was a crime until 1967 in England and Wales but by 2014 single‐sex marriages were recognised^17^. Single‐sex couples have access to IVF.

Alongside changes in family structure, there have been changes in conceptions of parenthood driven by both an increase in family breakdown and advances in reproductive technology. With increasing family breakdown and remarriage, families are constituted of parents and children who are not all biologically related.^18^ As well as this, reproductive technology exposes different roles to parenthood, allowing that a biological parent (the sperm or egg donor) need not be the parent raising the child. Macklin describes several different conceptions of families, amongst which are biological, legal, custom and subjective intentionality, concluding “…there is no single, univocal concept of the family…”.^19^ That there is not a stable recognized family structure undermines the claim that there can be a conception of family interests.

Those who argue for family interests could overcome the problem by stipulating a clear definition, but they don't. Ross’ conception is “…family is an intimate group in which the parent‐child relationship and its attendant obligations are central. This conception includes some legal families and excludes others. It also includes many non‐traditional, non‐legally sanctioned families. The intimate family is a moral and not a biological or legally defined relationship.”^20^ In excluding legal definitions of the family, family interests become impracticable for use in applied ethics because contentious decisions regarding family interests will go to law for resolution. Schoeman is similarly nonspecific “…‘family’ an intense continuing and intimate organization of at least one adult and child, wherein the child is extensively and profoundly dependent on the adult, in which the adult supplies the child with its emotional and material needs, and in which the parent is dependent on the child for a certain kind of intimacy. This relationship is to be understood as moral, not biological.”^21^ Some characterizations are almost deliberately obscure. For example the Nelsons write “…letting go of the idea that families *have* a defining essence…families as people configurations that have at least some of a rather wide array of characteristics, no one of which is definitive, but most of which will be present to one degree or another”.^22^ And from another author “…what is the ‘family’? As I will use it here, it will mean roughly ‘those who are close to the patient’…‘Family’ so defined will often include close friends and companions. It may also exclude some with blood or marriage ties to the patient.”^23^ Here Hardwig conflates friends and family. Sometimes friends are more knowledgeable about a person's values than are distant family members, but this does not mean that friends and family members should be confused. Taylor‐Sands recognizes the many different forms that families can take and settles on “…at the broadest level, a group of persons in a household who regard themselves as family.”^24^ This does not work for an infant, who cannot regard herself as a member of a family.

Through all of this there is no clear sense of “family”, which is needed by those who argue that family interests should play a role in medical decisions. An inconsistent approach to defining a family will produce an inconsistent conception of family interests. A first requirement from those who advocate family interests then is to be clear what they mean by “family”. Accepting that this is a problem to be overcome, as the concept of family is broadly accepted, the next question is whether a family is the sort of thing that can have interests.

### 2.2 Is the family the sort of thing that can have interests?

In this section, I argue that families can have interests in at least one sense. I distinguish a weak ‘collective’ conception of family interests from a strong ‘corporate’ conception of family interests.

People (or persons) uncontroversially have the highest moral standing, with morally significant interests. Because families are made up of people, one straightforward position is that family interests are the combined interests of family members and nothing more. Sumner argued “Any talk, therefore, of the welfare of groups, if it is not merely metaphorical, must be interpreted as referring to the aggregate of collective well‐being of their members. Collectivities have no interests to be furthered beyond those of individuals.”^25^ Buchanan and Brock described the claim for familial goals, perspective and objectives as “…dangerous reification”.^26^ List and Pettit endorse “‘normative individualism’…the view that something is good only if it is good for individual human beings….” arguing “…no approach we know suggests that group persons can have morally commanding interests such that a corporate good or benefit would determine what should be done, independently of the good or benefit to individuals”.^27^ I will call this amalgamation of individuals’ interests a weak conception of family interests.

There is a second, stronger, conception of family interests that families’ interests go above and beyond the interests of individual members of the family.^28^ As a school or sports club may have aims that are independent of the goals of the individuals who constitute the school or club, so too, some groups may thrive in a way that is not wholly dependent on the well‐being of individual members of the group. In this sense, family interests describe a communal interest not reducible to the individuals’ interests.^29^ In *Taking Families Seriously* the Nelsons state “Families aren't simply more or less efficient means to some independently specifiable good ends; they are also (at least oftentimes) valuable in themselves.”^30^ Ross states this position clearly “…families can have interests that are not reducible to the interests and needs of particular members…”.^31^

Either way, it is clear that in at least one sense the notion of family interests holds water and that the question is how should family interests be understood?

## 3 THE WEAK CONCEPTION

The weak, collective, conception is that family interests are no more than the combined interests of family members. All members of a family can be affected by decisions for one family member: “Decisions about a child's course of treatment affect not only the life and welfare of that child, but they often involve very significant financial, relational, and emotional consequences for the rest of the family”.^32^ Even for adults, Hardwig argued that an individual's medical interests (reflected by their autonomous choices) were only one, amongst many, factors that must be balanced “…the interests of patients and family members are morally to be weighed equally”.^33^ Similar criticisms can be mounted against an approach that prioritises the best interests of a child. Family interests “…replace the discreet and separable interests of family members with a more realistic view of the family, one that recognises the conflict, confluence and confusion of interests characteristic of life within a family”.^34^ Bainham argued for a “collective family interest” characterised as “…children are not just individuals, with individual interests. They are also members of a family unit and have an interest which forms part of the collective interests of that unit…There may also be a collective interest of the family (of which they are part) which needs to be taken into account…in some instances, the combined interests of the parents and the family taken as a whole may outweigh the interests of a particular child.”^35^ It may be that Bainham takes family interests to be the aggregate of the individuals’ interests. However, as he continues to argue “…we might need to throw in the desirability of preserving the family unit and holding it together if at all possible.”^36^ it may be that Bainham is arguing for a strong sense of family interests, over and above the aggregated interests of individual's (reinforced by his phrase ‘if at all possible’).

It is in an individual child's interests to grow up in an intimate family. A child in a flourishing family will probably have a better childhood and will be more likely to grow to be a healthy adult. So it is in any child's interests that some particular individual interests are set back to maintain family integrity. The family's value is instrumental to the child: The family itself is not intrinsically valuable. Even the weak conception of family interests justifies actions that may compromise an individual child's interests for benefits to others in the family.

An advantage of the weak conception of family interests is that it deals better with uncertainty about families than does the strong conception. The weak conception recognises family members as those whose interests are considered in amalgamated family interests. In situations where the make up of the family is disputed, anyone involved may deserve consideration when *overall* interests are calculated. Although there may be differences in the way that a person's interests are taken into account depending on whether or not they are taken to be a member of the relevant family, if a person plays a significant part in the life of a child, their interests deserve to be taken into consideration regardless of whether or not that person is part of the family.^37^ If so, whether a particular individual is, or is not, a member of the family may have little effect. My claim here is that if someone outside the family (a friend or a teacher perhaps) has played a significant role in the life of a child, then their interests deserve to be included, in some way, when decisions are made for a child.

Although this weak conception of family interests as the aggregated interests of individuals seems straightforward, important questions remain. Firstly, there needs to be a robust conception of an individual's interests, which we do not have.^38^ A second problem is that the way that individuals’ interests should be combined to generate the ‘collective’ family's interests remains to be settled. Smith suggests that “Each family member should be accorded maximal benefits relevant to his or her individual characteristics and compatible with maximal benefits relevant to others.”^39^ It is unlikely that any particular course will offer each individual *maximal* benefits and so there needs to be a balancing or optimising of the interests of the individual family members. Hardwig argues for “…the presumption of equality: The interests of patients and family members are morally to be weighed equally; medical and nonmedical interests of the same magnitude deserve equal consideration in making treatment decisions.”^40^ The balancing of interests is unlikely to be straightforward. Bainham described primary and secondary interests, reflecting the fact that some of our interests are more important than others, but there are more than two grades of interests. And even the same sorts of interests deserve different weights. For example frequent meals are more important for an infant than for an adult and so regular access to food is a more important interest for a baby. Balancing interests between family members is still more troubled because the balancing runs through time. A decision to prefer the interests of one child to another on a particular occasion need not be unfair, but it would be unjust if one child was consistently preferred to another (without good reasons). Whatever form of aggregation of interests is used, it is unlikely to be simple addition. Veatch asks “Surely, all that is expected is that a reasonable balance of the conflicting interests be pursued”.^41^ This is true, but the devil lies in the detail.

The third difficulty comes from the power imbalances within families and the extent of choice to conceptions of family interests. Given an objective conception of family interests construed as the combination of individuals’ interests, any individual (inside or outside the family) could determine the family's interests. However, as interests are usually taken to have a sizeable component of personal choice^42^ decisions about the family's interests rest at least partly in the family: The family must choose karate or music lessons (or neither). This provokes sufficient controversy when competent individuals choose their interests, but for family interests, there are more problems. We have already encountered the question of who counts as a member of the family. As well as this, there is a power imbalance within families: Parents make decisions concerning children, and will usually make the decisions that concern the family as a whole. This is trivially true for just‐born babies who can play no part in making decisions but it is also true for families with older children. In contentious decisions, the parents will usually make the final decision. A longstanding feminist criticism of the family is of the power imbalance within the family that leads to the interests of women being subordinated. Similarly in the absence of an objective notion of interests, and in the absence of oversight of decisions, a claim for family interests can become a claim that parents should make the decision, justified by whatever conception of interests (and whatever conception of a fair distribution of interests) the parents choose to use. The advantage that the weak conception of family interests has is that, as it is derived from the combined individuals’ interests, the individual's interests remain firmly in view in the reckoning of family interests.

## 4 THE STRONG CONCEPTION OF FAMILY INTERESTS

The second conception of family interests is that the family itself is the sort of group that has interests: “…families can have interests that are not reducible to the interests and needs of particular members…”.^43^ This echoes Schoeman's earlier writing “…the family is to be thought of as an intimate arrangement with its own goals and purposes”.^44^ Nelson described the importance of families as “…these particularities constitute how families distinguish themselves, how they become more than simply units of economic transfer. They express what might be regarded as familial character, as those reasons for which people have the deep and abiding interest they do in forming and maintaining families…”.^45^ Taylor‐Sands follows this in claiming “Intimate families are inherently valuable for the collective endeavour they entail, which gives our life meaning”.^46^ The Irish Constitution states “The State recognises the Family as the natural primary and fundamental unit group of Society, and as a moral institution possessing inalienable and imprescriptible rights, antecedent and superior to all positive law…”.^47^

The strong conception is that the family itself can thrive. The family is intrinsically valuable, over and above the benefits to individuals of being in a family. That the family delivers intimate relationships “…I want to get a clear account of the special moral value of intimacy squarely on the bio‐ethical table”^48^ is caught in the weak conception. The second, strong, conception describes that families are intrinsically valuable: “Seeing families and societies as institutions with independent ideals and as invested with meanings of their own is necessary to understanding what is so important about them…”.^49^

An example of the strong conception is that it can be used to make sense of talk about the interests of a group such as the British Royal Family. In the late 20^th^ Century, when the heirs to the throne were divorcing and attracting media attention for assorted misbehaviours. Prince Charles, the immediate heir, was disengaged from the problems of the times, and other members of the family were engaged in shady deals or partying with unwholesome individuals, it might be said that the Royal family was not thriving. The Royal Family itself (and not just its individual members) was failing. More recently, with handsome, photogenic heirs, spouses and babies, alongside members of the Royal Family who engage with the problems of our time (Prince Harry and his support for injured soldiers for example) and the Queen enjoying unrivalled popularity we could say that the Royal Family itself (regardless of the individuals themselves) is flourishing. The Royal Family is taken to represent a flourishing Britain, and to be successful itself. It is in this sense that there may be a strong conception of family interests.

There are several problems that need to be overcome to develop the strong conception of family interests. I will concentrate on three. Firstly, one question is whether the family is the sort of group to which it is appropriate to ascribe group interests.^50^ A concern is that the strong conception of family interests needs to recognise the tension between individual family member's interests and the family's interests. A second concern is that the dispute over family membership becomes a greater concern for the strong sense of family interests than the weak sense. A final concern is that the strong conception does not account for dysfunctional families.

### 4.1 Group Interests

The first question is whether a family is the sort of thing that can have interests that are over and above the interests of the individual members of the family? This seems possible for some groups: A sports team has a history, a personality (the Harlem Globetrotters), and common goals (to win at all costs, or to compete with style). But it doesn't make sense to talk of the interests of some other groups. The group of people waiting at a bus stop each have an interest in the bus taking them safely to their (different) destinations, but the group as a whole does not have a common interest over and above their individual interests.^51^ Does a family share the group characteristics of a sports club or a bus stop queue? Families are the sort of group for which it seems to make sense to talk about this sense of group interests. As the Irish Constitution makes clear, families are the building blocks of society. Families persist through time, members sharing interests in each other's interest and a common interest in the family. Family heirlooms and traditions are passed through generations. Family members are often devoted to others in the family and have pride in the achievements of family members and of the family as a whole. Parents *reproduce*, they do not just produce children.

Accepting these facts, families are different from the sorts of groups that are most studied.^52^ Individuals can join and leave other groups, whereas entry to, and exit from, family groups is constrained. Although adults may be able to join and leave family groups, children are not so empowered. Furthermore, in other groups for which group interests are plausibly claimed there are often structures and processes for decision‐making, which are characterised by transparency and governance. There may be routes of appeal for unpopular or seemingly‐wrong decisions. However in a family, there is an imbalance of power that is not found in many of the groups that can be considered to have group interests. Young children cannot contribute to decisions and older children's involvement may be limited. And the particular problem with the strong conception of family interests, is the sense that the family *has* to decide the family interests (analogously to the way that an individual's interests may have a large component of personal choice). Given this, a claim for strong family interests may become indistinguishable from a claim for strong parental authority.

Ross rejects this particular criticism arguing that “…parents perceive themselves as representatives of the family's interests, and this can be separated from their role as representatives of their own interests. As such parents can serve as both moderator and disputant in intimate family decisions.”.^53^ Ross’ claim that parents *can* stand above the family and take a more objective view of the decisions does not mean that parents *will* act in this way. In situations where a parent has to decide as both “moderator and disputant”, it is difficult for the parent to be objective, to compensate, but not over‐compensate. Buchanan and Brock describe conditions rebutting the presumptive decision‐making authority of the family for those incompetent to choose including “…the especially vulnerable position of the incompetent patient, the momentousness of the consequences of the decision…and some treatment alternatives would impose great burdens on the surrogate…”^54^ arguing for institutional review when they are present. Their conditions are true for all newborn and many older children. It is clear that Buchanan and Brock did not intend to refer to normal children, but as babies are amongst the most vulnerable of humans, and bringing up a child holds enormous burdens for the parents (alongside great rewards), their conditions emphasize the need for caution.

Ross’ second response to the imbalance of power within families is that it “…ignores the influence that children, even infants, have in eliciting responses in adults that can profoundly influence relationship and goals. Although parents have ultimate authority, their decisions are influenced by the needs and interests of children. Parental decisions can reflect a family decision.”.^55^ Ross’ second claim is that children *can* influence the family decision. Again the response to Ross is to agree that some children can, on some occasions, influence some parents. This does not mean that a child is guaranteed the consideration that they deserve.

These are good reasons why the claim that the family can be the sort of group that can hold group interests as anything other than the amalgamation of the interests of the individual members of the group should be dismissed. A second problem with strong conceptions of family interests is that families are not clearly defined.

### 4.2 The lack of a clear definition of the family and the strong conception

A clear definition of the family is a more important requirement for the strong conception of family interests than it is for the weak conception of family interests. This is so because those who are responsible for selecting the values that underpin the strong conception of family interests and so will then be bound by family values must be clearly identified. The strong conception of family interests is undermined if family members are bound by values that they had played no part in selecting. Infants and young children can have no say in the selection of family values and interests. Even accepting this, the internal power structure of at least some families will mean that not all family members who are able are offered an appropriate say in choosing the family values. The vagueness that can be tolerated to the borders of the family for the weak conception will not work for the strong conception.

### 4.3 Dysfunctional families

Much of the literature on family interests concentrates on the advantages of families. Families are taken to be an undiluted good. As examples, “…family members cherish each other simply for each other's sake, and that being devoted to 'the family' and its members is a source of deep meaning and value in our lives and the lives of those around us”^56^ and “Intimate families are inherently valuable…which gives our life meaning”.^57^ Although families are often a source of support, and both material and emotional sustenance and of great value to the individual members, this is not always true.^58^ Some families are clearly dysfunctional and harm at least some of their members. Children (and adults) are neglected, emotionally abused, injured and killed in intimate families.^59^ Dysfunctional families are the subject of much literature (amongst which are *Snow White*, *Oranges are not the only Fruit, Why be Happy When You Could be Normal?* and *the Goldfinch* as examples) and many films (*Mommie Dearest*, *the Sopranos* and *Shameless* are amongst many others). A theory that accounts for family interests also needs to recognise and account for dysfunctional families. Families cannot be taken to be a source of unmitigated good. How does the strong conception of the family deal with these families?

The concern is that claims for a strong conception of family interests means that an individual's interests may not be apportioned proper weight. This is particularly problematic when dealing with young children, but can be true even for adults. And if the strong conception of family interests is preferred, it becomes more difficult for those outside the family to enquire into situations in which children (and adults) are mistreated or neglected.^60^

I have argued that there are at least two sense of family interests, and that there are unresolved concerns with both of them. I will now argue that the weaker sense of family interests is more plausible, but even so family interests should not contribute to the analysis of contested medical decisions for children.

## 5 SHOULD FAMILY INTERESTS CONTRIBUTE WHEN DECIDING MEDICAL INTERVENTIONS FOR CHILDREN?

On some occasions, parents must make decisions that are not in a particular child's interests, but are for the good of others in the family.^61^ These decisions cannot be justified by the individual's interests, and can only be justified by an appeal to the interests of others, or to family interests. The weak conception of family interests justifies actions that at first sight are against an individual person's interests, but when the benefits – to the child – of being in a family are included, they are in the child's *overall* interests. As well as this, the weak conception of family interests allows the interests of others within the family to be considered to justify decisions that are not in a particular child's interests “To be part of a family is to be morally required to make decisions on the basis of thinking about what is best for all concerned, not simply what is best for yourself.”^62^

This is true, but as Munoz‐Darde argues “…justice requires us not to be concerned with family welfare or autonomy, but with each family member's demands for respect and well‐being.”^63^ The risk is that “…if we give moral standing to groups as such, we shall lose sight of individuals within the group”^64^. And this is a particular concern for children, because of their vulnerability and their difficulty in making their voices heard.^65^ Adults are the voice for young children. Even older children may struggle to be heard. The concern is that claims for family interests can “…reinforce the power of conservative elites whose wishes and interests clash with those of others in the group. Typically an elite will want to use its power to maintain the traditions and integrity of the group and will be unwilling to tolerate dissent, deviance and demands for reform.”^66^. The weaker conception of family interests (the amalgamated interests of individual family members) is more plausible as the interests of the individual members of the family remain visible as the components that are considered in coming to a decision about family interests. This is not true of the strong conception of family interests.

Despite the stronger position of the weak conception, it is not clear that family interests can contribute to the analysis of contested medical decisions. MB was an infant with SMA (spinal muscular atrophy, a lethal, inexorable, degenerative neuromuscular disease presenting in infancy). MB's parents wanted aggressive treatment to continue but the clinical team argued for palliation.^67^ The judge decided that it was in MB's objective best interests that treatment should continue. Inwald recommends the “family‐based welfare approach” which is “…understood as we understand children's lives in the context of their family”^68^ to resolve this case. But it is not clear that a family‐based welfare approach adds anything. If it was in MB's interests that treatment should continue, is it obvious that a detrimental effect of continued treatment on family welfare (perhaps manifest as inconvenience to his parents, or that MB's parents distraction to their ill child is against the siblings’ interests or both) would mean that MB's treatment should be discontinued? Conversely, if the likelihood of success of treatment combined with the unpleasantness of the treatment are such that it is clear that treatment is not in MB's interests, is it obvious that if the family's welfare is in favour of continuation of treatment (perhaps his parents’ and siblings’ love for him is such, or that his family cannot bear the thought of MB's death) that treatment should continue? In neither case is it clear that family interests should override what would be in MB's interests, and the conclusion that family interests would override an individual's interests (in either way) is highly counterintuitive. I believe that the individual interests of others may be included when medical decisions are made for children (with a “reasonable” assessment of the individual child's interests replacing the child's best interests). But the addition of a separate component of family interests (if we can be clear about what is meant by “family interests”) adds complexity without aiding resolution. Other individuals’ interests may be considered, but these are included as *individual* interests not as an entity of family interests.

## 6 CONCLUSION

I have argued that that family interests are neither clear, nor agreed, and that those who appeal to family interests have not made their meanings clear.^69^ I have argued that only a weak conception of family interests (as the aggregate of the individual family members’ interests) is plausible. The weaker conception justifies some actions that do not benefit an individual child at first sight, firstly for advantages that accrue to the child herself in remaining in her family, and secondly for advantages to others in the family (because of justice). However, a tension remains between the individual child's interests and the interests of others in the family, limiting the usefulness of family interests in influencing medical decisions for a child.

## Footnotes

1
Amongst examples are, “Doctors should always act in the best interests of children and young people.” The General Medical Council. (2007). *0–18 years: Guidance for all doctors* Retrieved from http://www.gmc-uk.org/0_18_years___English_0911.pdf_48903188.pdf; “Above all…placing the child's best interests at the center of all clinical considerations.” Royal College of Paediatrics and Child Health. (1999). *Duties of a Paediatrician* Retrieved from http://www.rcpch.ac.uk/system/files/protected/page/Duties%20of%20a%20paediatrician.%201999.pdf. “…the best interests of a baby must be a central consideration in determining whether and how to treat him or her.”; Nuffield Council on Bioethics. (2006). *Critical Care Decisions In Fetal And Neonatal Medicine: Ethical Issues* xvii. “…the child's welfare shall be the court's paramount consideration.” The Children Act (1989). Retrieved from http://www.legislation.gov.uk/ukpga/1989/41/data.pdf. “The matter must be decided by…the best interests of the patient” *An NHS Trust v MB* [2006] EWHC 507 (Fam) paragraph 16. “…we apply a child's best interest standard.”; Schuklenk, U. (2012). Europe debates circumcision … and what about the child's best interest. *Bioethics*, *26*(8), iii. 10.1111/j.1467-8519.2012.02010.x22971182

2
I will use “interests”, “welfare” and “well‐being” interchangeably as other have.

3
For example “…while the BIS [best interests standard] has potent rhetorical power, its invocation and application is actually quite inconsistent, and that ultimately, it should be rejected”. Salter, E. K. (2012). Deciding for the child: A comprehensive analysis of the best interest standard*. Theoretical Medicine and Bioethics*, *33*(3), 180. 10.1007/s11017-012-9219-z22528148

4
Amongst examples: “…allows them [the parents] to act for the well‐being of the family…When the self‐regarding interests and goals of a child conflict with the group goals of the family, the parents may compromise the interests of the child…” Ross, L. F. (1998). *Children, families and health care decisions* (pp. 44). Oxford University Press: Oxford; “It has been suggested that physicians should take a greater role in encouraging and supporting family‐centered decision‐making…” Hardart, G. E., & Chung, W. K. (2014). Genetic testing of children for diseases that have onset in adulthood: The limits of family interests. *Pediatrics, 134*, S105; “…familial considerations must be taken into account in medical decision‐making, and that sometimes these considerations have a greater claim on health care professionals than do the best interests of the pediatric patient” Lindeman, H. (2014). Why families matter. *Pediatrics 134,* S98. “…it is permissible to put the interests of the family above those of the infant…”; Strong, C. (1984). The neonatologist's duty to parents and patients. *The Hastings Center Report 4*(4), 13. “The aim of intensive care should be to treat the family, not just the patient.”; Inwald, D., Jakobovits, I., & Petros, A. (2000). Brain stem death: Managing care when accepted medical guidelines and religious belief are in conflict. *British Medical Journal, 320,* 1266–1267. And there are empirical claims that this is what does happen in practice: “An approach that considers family welfare rather than purely best interests of an individual child is a model that is used by the majority of paediatricians; Larcher, V., Craig, F., Bhogal, K., Wilkinson, D., Brierley, J. (2015). On behalf of the royal college of paediatrics and child health. Making decisions to limit treatment in life‐limiting and life‐threatening conditions in children: A framework for practice. *Archives of disease in childhood*, *100*(Suppl 2), s11; “…there is broad recognition that families routinely make decisions that consider communal family interests…”; Hardart & Chung, *op. cit* note 4, p. S105. And in adult critical care “…a majority of the physicians surveyed believed that…family interests should be an important consideration in medical decision making for incompetent patients”; Hardart, M. K. M., & Truog, R. D. (2003). Spinal muscular atrophy–type I. *Archives of Disease in Childhood, 88*, 1899.

5
Ibid: 850, “The greatest challenge is anticipating what each family would consider adequate quality of life for their child”.

6
“The aim of intensive care should be to treat the family not just the patient…it is more important to respect the cultural traditions of the family…It should be possible to reach a compromise with the religious authorities…”, see Inwald et al., *op. cit*. note 4, p. 1268.

7
List, C., Pettit, P. (2011). *Group Agency: The possibility, design, and status of corporate agents* Oxford University Press; Jones, P. (1999). Group rights and group oppression. *The Journal of Political Philosophy, 7*(4), 353–377; Jones, P. (2014). Group Rights. In E. N. Zalta (Ed.), *The Stanford Encyclopedia of Philosophy* (Spring Edition). Retrieved from http://plato.stanford.edu/archives/spr2014/entries/rights-group/; Tollefsen, D. P. (2015). *Groups as agents*. Polity Press.

8
Strong, *op. cit*. note 4, p. 10.

9
Draper, H. (2013). Grandparents' entitlements and obligations. *Bioethics, 27*(6), 309–316. 10.1111/bioe.12028PMC377095823718643

10
See for examples: Inwald, D. (2008). The best interests test at the end of life on PICU: A plea for a family centred approach. *Archives of Disease of Childhood, 93*, 248–250; Gopfert, M., McClelland, N., & Wilson, J. (2010). Maternal mental health: An ethical base for good practice. In D. Kohen (Ed.), *The Oxford Textbook of Women and Mental Health* (Chapter 8). Oxford University Press: Oxford. 10.1136/adc.2006.11112017932122

11
Nelson describes “They express what might be regarded as familial character…For instance, a family may encourage and support music lessons but not karate lessons…” Nelson, J. L. (1992). Taking families seriously. *The Hastings Center Report, 22*(4), 8.

12
Recognizing that this is a difficult and contested area, see Wiggins, D. (1994). Objective and subjective in ethics, with two postscripts about truth. *Ratio (new series), VIII 7 December*, 243–258.

13
Nussbaum, M. (2006). *Frontiers of justice disability, nationality, species membership: Disability, nationality, species membership (Tanner lectures on human values)* (pp. 105–106). First Harvard University Press.

14
An Australian Government report into adoptions between 1950 to 1992 reported “Attitudes towards the women…resulted in feelings of shame, guilt and an unworthiness to raise their child.” Kenny, P., Higgins, P.D., Carol Soloff, C., & Sweid, R. (2012). *National research study on the service response to past adoption practices* Research Report No. 21 — August, Chapter 5. Retrieved from http://www.aifs.gov.au/institute/pubs/resreport21/: 35. Other examples of mothers’ experiences from the Australian national research study of past adoption practices include “I was only 17 and society did not accept unwed mothers…I was told, being unmarried, I wasn't fit to be a mother…Being locked up in an institution with no choice, no support, and treated like I was nothing.” Ibid: 35. These attitudes were widely reported, for example in Ireland, Interdepartmental Committee to establish the facts of State Involvement with Magdalen Laundries. (2013). chapter 3. Retrieved from http://www.idcmagdalen.ie/consulted 20.6.2, chapter 3; in Britain, Paton, M. (2012). *Sin and the Single Mother* *The Independent* 26 May. Retrieved from http://www.independent.co.uk/news/uk/this-britain/sin-and-the-single-mother-the-history-of-lone-parenthood-7782370.html; in America, Wilson‐Buterbaugh K. (2013). *Not by Choice* Retrieved from http://www.eclectica.org/v6n1/buterbaugh.html

15
Adams, S., Rainey, S., & Beckford, M. (2011). *Single women being offered IVF on the NHS* *The Telegraph* Retrieved from http://www.telegraph.co.uk/women/womens-health/8844762/Single-women-being-offered-IVF-on-the-NHS.html

16
“The London Women's Clinic has always been a popular centre for single women because of our large sperm bank and our welcoming attitude towards alternative families. The seminars focus on the various fertility treatments for single women…” The London Women's Clinic. *Open Days* Retrieved from http://www.londonwomensclinic.com/index.php/london/open-days

17
Marriage (Same sex couples) Act 2013. Retrieved from http://www.legislation.gov.uk/ukpga/2013/30/contents/enacted/data.htm

18
An example of the challenges that result comes from the US courts: A 13 year old boy with leukemia needed a bone marrow transplant. He shared a father with 3 year old twins (born to a different mother). The twins lived with their mother, not their father. There had been infrequent contact between the 13 year old and the twins. Having failed to persuade the twin's mother, their father sought a court order to compel testing and bone marrow donation if the twins were compatible: Curran v. Bosze, 141 Ill. 2d. 473, 566 N. E. 2d. 1319 (1990). The court withheld permission. The case gives a clear example of the complexities of modern day families. How would family interests be understood in this case?

19
Macklin, R. (2007). *Artificial means of reproduction and our understanding of the family* In H. La Follette (Ed.), *Ethics in Practice* (Chapter 20, p. 11). Blackwell.

20
Ross, *op. cit*. note 4, p. 6.

21
Schoeman, F. (1980). Rights of children, rights of parents, and the moral basis of the family. *Ethics*, *91*(1), 9–10.

22
Nelson, H. L., & Nelson, J. L. (1995). *The patient in the family: An ethics of medicine and families* (p. 35; Nelsons’ italics). Routledge.

23
Hardwig, J. (1990). What about the family? *The Hastings Center Report, 20*(2), 5. 2318632

24
Taylor‐Sands, M. (2010). Saviour siblings and collective family interests. *Monash Bioethics Review*, *29*(2), 12.4. 10.1007/BF0335152222032019

25
Sumner, L. W. (1996). *Welfare happiness and ethics* (p. 179). Clarendon Press Oxford.

26
Buchanan, A. E., & Brock, D. W. (1990). *Deciding for others: The ethics of surrogate decision making* (p. 236). Cambridge University Press.

27
List & Pettit, *op. cit*. note 7, p. 182.

28

*op. cit* note 4. Schoeman, F. (1985). Parental discretion and children's rights: Background and implications for medical decision‐making. *The Journal of Medicine and Philosophy 10*, 45–61; Nelson, *op. cit* note 11, pp. 6–12; Taylor‐Sands, *op. cit*. note 24, pp. 12.1–12.15. 10.1093/jmp/10.1.453981082

29
Jones draws a similar distinction when considering group rights “*…corporate* will be used here to describe…a right‐holding group as a unitary entity. The term *collective* will be used to describe the conception of a group right as a shared or joint right, since it conceives a right‐holding group as a ‘collection’ of individuals.” Jones, *op. cit*. note 7, Jones’ italics.

30
Nelson, *op. cit*. note 22, p. 7.

31

*op. cit*. note 4, p. 43.

32
Salter, *op. cit*. note 3, p. 183.

33
Hardwig, *op. cit*. note 23, p. 7.

34
Crouch, R., Elliott, C. (1999). Moral agency and the family: The case of living related organ transplantation. *Cambridge Quarterly of Healthcare Ethics, 8*: 284. 10.1017/s096318019980303x10388928

35
Bainham, A. (1998). Honour thy father and thy mother: Children's rights and children's duties. Chapter 6. In G. Douglas and L. Seba (Eds.). *Children's rights and traditional values* (pp. 99). Dartmouth.

36
Ibid: 102.

37
The extent to which partiality can be shown to those in the family over those outside the family is disputed “Family loyalties present two sources of potential conflict…and extra weight given to the promotion of the family either at the expense of those outside the family or even at the expense of those within”. Schoeman, *op. cit*. note 28, p. 54.

38
Griffin, J. (1986). *Well‐being: Its meaning measurement and moral importance* Oxford University Press; Degrazia, D. (1995). Value theory and the best interests standard. *Bioethics, 9*(I), 50–61. 10.1111/j.1467-8519.1995.tb00300.x11653032

39
Smith, P. (1993). Family responsibility and the nature of obligation. In D. Tietjens Meyers., K. Kipnis, & C. F. Murphy, Jr. (Eds.). *Kindred matters; rethinking the philosophy of the family* (p. 49). Cornell University Press.

40
Hardwig, *op. cit*. note 23, p. 7.

41
Veatch, R. M. (1995). Abandoning informed consent. *The Hastings Center Report*, *25*(2), 7 7782200

42
The British Medical Association goes further than most in stating “Competent adults are allowed to define their own concept of ‘best interests’, even if their views are very different from those of the rest of society.” Ethics Department BMA. (2004). *Medical Ethics Today: The BMA's Handbook of Ethics and Law. 2nd Edition* (p. 136). BMJ Publishing Group; More usually a component of personal choice is recognised. Scanlon writes of well‐being that “…adequate criteria must allow for the fact of individual variation in taste and interest. (I leave aside for the moment consideration of the various ways in which this might be ‘allowed for.’)” Scanlon, T. M. (1975). Preference and urgency. *The Journal of Philosophy LXXII no 19 November 6,* 655.

43

*op. cit*. note 4, p. 43.

44
Schoeman, *op. cit*. note 28, p. 50.

45
Nelson, *op. cit*. note 11, p. 8.

46
Taylor‐Sands, *op. cit*. note 24, p. 12.7.

47
Constitution of Ireland. (1937). Available at http://www.irishstatutebook.ie/en/constitution/index.html: Article 41

48
Nelson, *op. cit*. note 11, p. 7.

49
Schoeman, *op. cit*. note 28, p. 56.

50
I've argued that the British Royal Family may have group interests, but this may be a particular case. It may be that families in general do not have group interests, but the British Royal Family has group interests as a ruling monarchy, not as a family.

51
Jones, *op. cit*. note 7.

52
Tollefsen's groups are *corporate* groups “…paradigm case of a corporate group is a corporation, but governments, educational institutions will also count as corporate groups insofar as they have a structure and decision‐making process.” Tollefsen, *op. cit* note 7, p. 3. For List and Pettit group agents have representational states, motivational states, and can process these so it can “…intervene suitably in the environment…” see, List & Pettit, *op. cit*. note 7, p. 20.

53

*op. cit* note 4, p. 32. Others make similar claims “Parents can be seen as representing the interests of the family as an integrated whole in addition to representing their own particular interests.” Schoeman, *op. cit*. note 21, p. 19.

54
Buchanan & Brock, *op. cit*. note 26, pp. 142–143.

55

*op. cit*. note 4, p. 33.

56
Crouch & Elliot, *op. cit*. note 34, p. 283.

57
Taylor‐Sands, *op. cit*. note 24, p. 12.7.

58
Larkin expressed the concern that families are dysfunctional somewhat pessimistically “They fuck you up, your mum and dad./They may not mean to, but they do./They fill you with the faults they had/And add some extra, just for you.” Larkin, P. (2001). *This be the Verse* In A. Thwaite, (Ed.). *Collected Poems*. Farrar, Strauss and Giroux.

59
Researchers covering the whole of Scotland (for 18 months) state “We identified 19 cases of NAHI [Non‐accidental head injury], 12 boys and seven girls, between July 1, 1998 and December 31, 1999. The annual incidence of NAHI was 24·6 per 100 000”. Barlow, K. M., & Minns, R. A. (2000). Annual incidence of shaken impact syndrome in young children. *The Lancet, 356*(9241), 1571. 10.1016/S0140-6736(00)03130-511075773 There are high profile tragic cases where children have been killed by their parents, or those their parents have entrusted their children to, described in various reports including: Lord Laming. *The Victoria Climbie Inquiry Report* 2003. Retrieved from http://webarchive.nationalarchives.gov.uk/20130401151715/ https://www.education.gov.uk/publications/eOrderingDownload/CM-5730PDF.pdf; Haringey Local Safeguarding Children Board. (2009). *Serious case review: Baby peter. Executive summary* Retrieved from http://www.haringeylscb.org/executive_summary_peter_final.pdf; Coventry Safeguarding Children Board. (2013). *Serious Case Review re Daniel Pelka* Retrieved from http://www.coventrylscb.org.uk/files/SCR/FINALOverviewReportDP20130913%20Publication%20version.pdf And adults are mistreated too: “One woman in four (and one man in six) in the UK will be a victim of domestic violence during their lifetime, according to research estimates. Two women a week are killed by a current or former male partner” NHS Choices. Getting Help for Domestic violence. Retrieved from http://www.nhs.uk/Livewell/abuse/Pages/domestic-violence-help.aspx. van Eck, C. (2003). *Purified by Blood: Honour Killings amongst Turks in the Netherlands* Amsterdam University Press. Retrieved from http://www.google.co.uk/url?url= http://www.oapen.org/download%3Ftype=document%26docid=340240&rct=j&q=&esrc=s&sa=U&ei=fyZOVJ7yEafY7Ab5i4GwAg&ved=0CBQQFjAA&usg=AFQjCNE0GEq8_oiquWhgmVv5K5eXA-oLYw

60
In some abusive families, the child may be reluctant to disclose abuse for fear of consequences (including scrutiny from outside the family, the disruption of familial relationships and punishment of abusive members). And when the child is taken into care she may fear that these fears are fulfilled and she is scapegoated.

61
“Just as the interests of the interests of the infant limit parental authority, so the interests of the family limit what can be required of the family for the sake of an infant” , see Strong, *op. cit*. note 4, p. 15.

62
Hardwig, *op. cit*. note 23, p. 6.

63
Munoz‐Darde, V. (1999). Is the family to be abolished then? *Proceedings of the Aristotelian Society*, *99,* 39.

64
Jones, *op. cit*. note 7.

65
An example of this – though it is more usually phrased as a right to religious freedom, or a right to parental autonomy than as a family interest – is found in the USA. The majority of states have an exemption to parental prosecution for neglect if the neglect was motivated by the parents’ religious belief (such as Christian Science) Asser, S. M., & Swan, R. (1998). Child fatalities from religion‐motivated medical neglect. *Pediatrics, 101*(4 Pt 1), 625–629. 10.1542/peds.101.4.6259521945

66
Jones, *op. cit* note 7. As an example, Van Eck describes honour killings in immigrant communities in the Netherlands. “Honour killing is the most extreme solution: It only becomes an option when the alternatives have failed and the question of honour escalates. The reason that honour killings do not occur more frequently is that people do their utmost to prevent questions of honour from arising” van Eck, *op. cit*. note 59, p. 185.

67

*An NHS Trust v MB* [2006] EWHC 507 (Fam).

68
Inwald, *op. cit*. note 10, p. 250.

69
Wilkinson makes a similar point in arguing against a family veto over posthumous organ donation, “…it is hard to defend this view in the absence of a detailed case for family autonomy. It is really up to someone who wishes to defend a significant role for the family to make this case.” Wilkinson, T. (2007). Individual and family decisions about organ donation*. Journal of Applied Philosophy*, *24*(1), 36.

